# Ginsenoside Compound K Ameliorates Development of Diabetic Kidney Disease through Inhibiting TLR4 Activation Induced by Microbially Produced Imidazole Propionate

**DOI:** 10.3390/ijms232112863

**Published:** 2022-10-25

**Authors:** Qian Chen, Dongwen Ren, Luokun Liu, Jingge Xu, Yuzheng Wu, Haiyang Yu, Mengyang Liu, Yi Zhang, Tao Wang

**Affiliations:** 1 State Key Laboratory of Component Based Chinese Medicine, Tianjin University of Traditional Chinese Medicine, 10 Poyanghu Road, Jinghai District, Tianjin 301617, China; 2 Haihe Laboratory of Modern Traditional Chinese Medicine, Tianjin University of Traditional Chinese Medicine, 10 Poyanghu Road, Jinghai District, Tianjin 301617, China

**Keywords:** gut microbiota, ginsenoside compound K, imidazole propionate, TLR4, diabetic kidney disease

## Abstract

Diabetic kidney disease (DKD) is a common and devastating complication in diabetic patients, which is recognized as a large and growing problem leading to end-stage kidney disease. As dietary-mediated therapies are gradually becoming more acceptable to patients with DKD, we planned to find active compounds on preventing DKD progression from dietary material. The present paper reports the renoprotective properties and underlying mechanisms of ginsenoside compound K (CK), a major metabolite in serum after oral administration of ginseng. CK supplementation for 16 weeks could improve urine microalbumin, the ratio of urinary albumin/creatinine and renal morphological abnormal changes in db/db mice. In addition, CK supplementation reshaped the gut microbiota by decreasing the contents of *Bacteroides* and *Paraprevotella* and increasing the contents of *Lactobacillu* and *Akkermansia* at the genus level, as well as reduced histidine-derived microbial metabolite imidazole propionate (IMP) in the serum. We first found that IMP played a significant role in the progression of DKD through activating toll-like receptor 4 (TLR4). We also confirmed CK supplementation can down-regulate IMP-induced protein expression of the TLR4 signaling pathway in vivo and in vitro. This study suggests that dietary CK could offer a better health benefit in the early intervention of DKD. From a nutrition perspective, CK or dietary material containing CK can possibly be developed as new adjuvant therapy products for DKD.

## 1. Introduction

Diabetic kidney disease (DKD), also known as diabetic nephropathy, is a kind of glomerulosclerosis induced by long-term diabetic metabolism disorder, which is recognized as a predominant risk factor for end-stage renal disease (ESRD) in adults. In general, thickening of the glomerular and tubular basement membrane can be found in patients who have had type 2 diabetes for just 1.5–2.5 years, with clinical manifestation of intraglomerular hypertension, glomerular hypertrophy, and glomerular hyperfiltration [1]. An increase in glomerular mesangial area can occur after 4–5 years of diabetes progression, with elevation of the ratio of the mesangial matrix from the normal 20% to 40%. The high intraglomerular pressure induces an increase in pore size in the glomerular basement membrane, which leads to microalbuminuria [2,3].

When the ratio of mesangial matrix increases to 60–80%, the degree of kidney damage reaches stage 3 of chronic kidney disease (CKD), at which the glomerular filtration rate is less than 60 mL/min/1.73 m^2^, indicating that half of the kidney function has been lost [2,4,5]. Although blood glucose control and blood pressure control slow the progression of albuminuria to some extent, the majority of the patients still develop DKD later, and eventually renal failure and ESRD [6,7]. Therefore, it has long been accepted that DKD-related damage is irreversible.

The underlying pathophysiological mechanism of DKD remains unclear, which makes it difficult to clinically control the DKD initiation and progression. Recent research has suggested that gut microbiota plays an important role in the development of DKD [8]. The intestinal bacteria are involved in the hemodynamics, lipid and glucose metabolism, endotoxin accumulation, and inflammation reactions to affect the onset and prognosis of DKD [9,10].

A long-term hyperglycemic state triggers the imbalance of intestinal bacteria and increases the permeability of the gut barrier. The metabolites of harmful bacteria pass through the gut barrier and enter circulation, together with the hyperglycemia-related abnormal metabolism of glucose, lipid, and protein, and induce injury to other organs [11]. For example, accumulations of gut microbe-derived metabolites (trimethylamine *n*-oxide, bile acids, phenyl sulfate), in the kidneys cause oxidant stress, endoplasmic reticulum stress, inflammation, and fibrosis, exacerbating kidney injury and albuminuria in DKD [12,13,14,15].

Unbalanced gut microbiota in DKD is basically caused by the unfavorable intestinal microenvironment induced by a long-term hyperglycemic condition, which is impossible to be recovered by the administration of probiotics [16]. To our knowledge, there is no clinical evidence to support that probiotic treatment is beneficial to DKD to date. Moreover, using probiotics carries potential unpredictable risks in DKD patients with a hyperpermeable intestinal barrier.

On the other hand, protein is one of the essential elements for maintaining basic functions and processes in humans. Therefore, excessive protein restriction is not advised for the prevention of early renal function decline [17]. We prefer the regulation of intestinal flora and reducing pathogenic effects of endotoxin as possible effective managing strategies for DKD.

Recently, active compounds from herbal dietary supplements have been widely used for DKD [18]. These compounds have been reported to regulate the intestinal microenvironment and protect renal function, and are considered as protective adjuvant therapy for diabetes and DKD. In East Asia, *Panax Ginseng* is one of the commonly used traditional medicines with positive effects on DKD [19,20]. In Europe and America, ginsenosides has been used as a dietary supplement to improve immunity and prevent metabolic diseases such as diabetes [21,22,23].

In this paper, we investigated the potential effects of CK on the development of DKD in mice. We studied the therapeutic effects and mechanisms of CK on renal function declining from different aspects including renal pathophysiology, intestinal flora composition, and protein expression. In addition, in order to improve the quality of life of DKD patients, from a nutrition perspective, we investigated the possibility of developing new adjuvant therapy products from ginseng for early-intervention and prevention of DKD.

## 2. Results

### 2.1. CK Supplementation Protects against Diabetic Kidney Injury in db/db Mice

**Figure 1:**
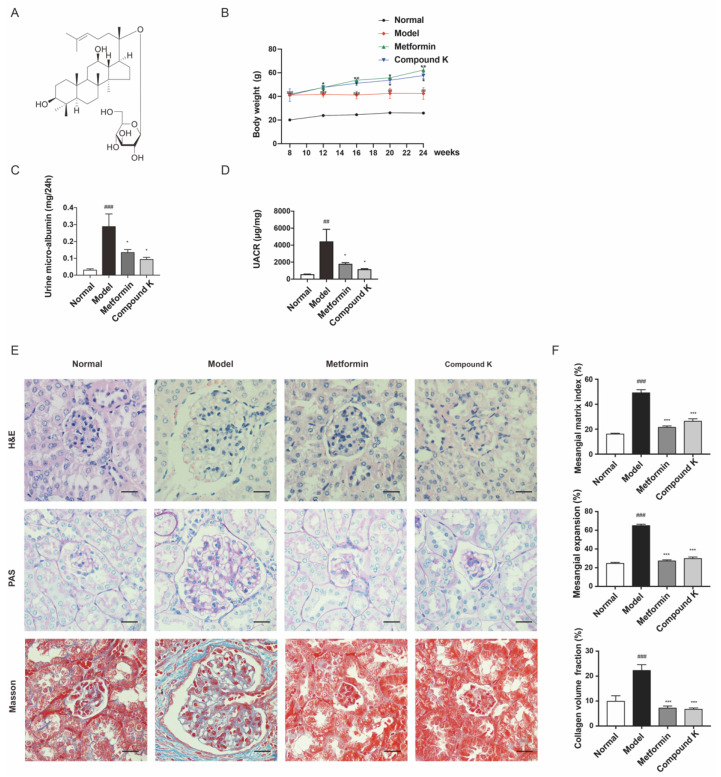
CK supplementation protects against diabetic kidney injury in db/db mice. (**A**) Chemical molecular structure of CK. (**B**) Body weight of experimental mice. (**C**,**D**) Levels of urine microalbumin and UACR. (**E**) HE staining was used for analysis of histological abnormalities, PAS staining was used for the detection of glycogen (purple), and Masson staining was used for the detection of type Ⅳ collagen (blue) in kidney section. Scale bar, 20 μm. (**F**) Mesangial matrix index of 20 randomly selected glomeruli per mouse in each group (HE); semiquantification of mesangial expansion (PAS) and collagen volume fraction (Masson) in the glomeruli of the different groups. Data were expressed as means ± SEM (*n* = 7). ^###^ *p* < 0.001, ^##^ *p* < 0.01 compared to normal. *** *p* < 0.001, ** *p* < 0.01, * *p* < 0.05 compared to model.

In this study, an obvious increase in body weight was shown in db/db mice supplemented with CK and metformin, compared with the model group (Figure 1B). After 16 weeks, renal function injury was evidenced by an increase in urine microalbumin (Figure 1C) and UACR (Figure 1D) in db/db mice. However, CK supplementation attenuated proteinuria significantly, similarly with metformin. Moreover, histopathological analysis of the glomeruli area stained with HE, PAS and Masson demonstrated an increase in glomerulus expansion, collagen accumulation, glomerulosclerosis, the narrowing of capillary lumen, the size of diffused matrix, and peripheral capillaries of thick stiff wall (Figure 1E,F). These data exhibited that CK supplementation can slow the progression of DKD in db/db mice.

### 2.2. CK Supplementation Modifies the Diabetic Mice Gut Microbial Composition to Modulate Renal Function

**Figure 2:**
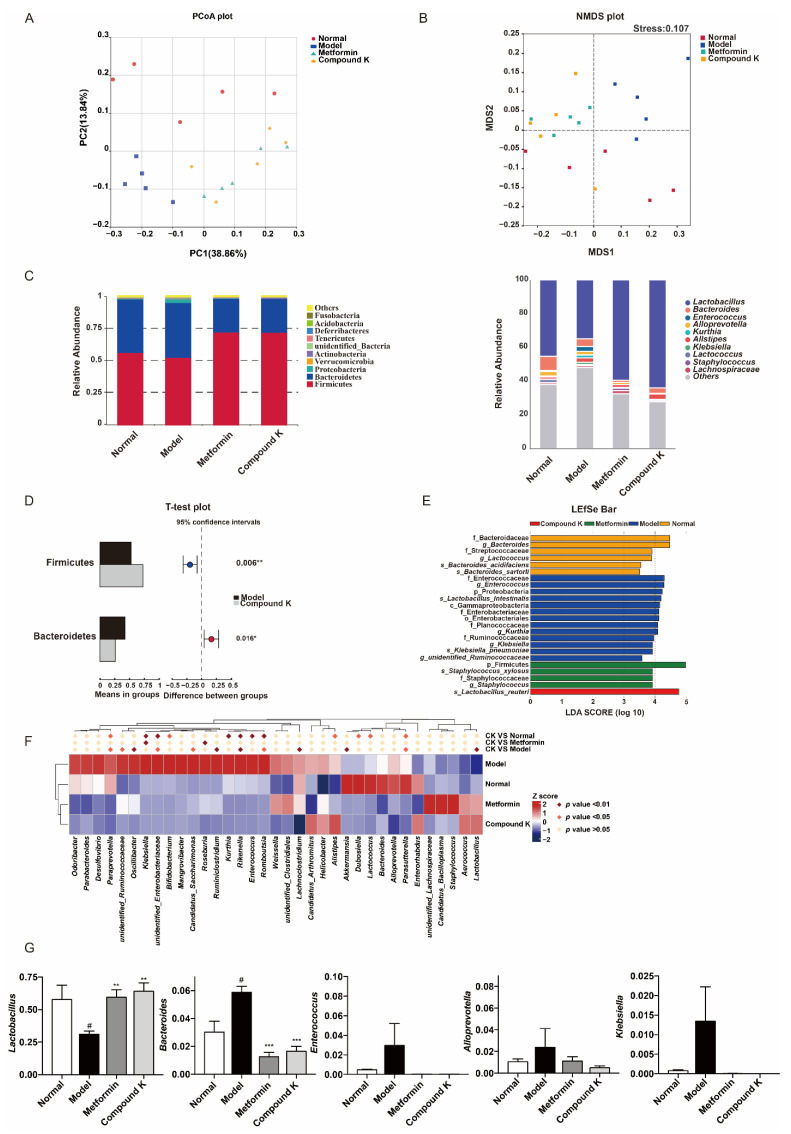
CK supplementation modifies the diabetic mice gut microbial composition to modulate renal function. (**A**) The difference of gut microbiota was analyzed in a principal coordinates analysis (PCoA) plot. The two principle coordinates (PC1-PC2) were 38.86% and 13.84%, respectively. (**B**) The difference in gut microbiota was analyzed in non-metrical multidimensional scaling (NMDS). The stress was 0.107. (**C**) Comparison of gut microbiota composition in four experimental groups at phylum and genus level. (**D**) The relative abundance of phylum Firmicutes, and Bacteroidetes between model and CK supplementation groups, shown in histogram. (**E**) Different taxa of gut microbiota in four experimental groups detected in LefSe analysis. (**F**) Heatmap showed the top 35 abundant microbes at genus level (log_10_ transformation). The comparison was determined by *p* value between two different groups using the proportions of genus in gut microbiota of each group. (**G**) The relative abundance of *Lachnoclostridium*, *Bacteroides*, *Enterococcus*, *Alloprevotella*, and *Klebsiella* between all groups, shown in histogram. Data were expressed as means ± SEM (*n* = 5). ^#^ *p* < 0.05 compared to normal. *** *p* < 0.001, ** *p* < 0.01, * *p* < 0.05 compared to model.

Generally, as reported previously, compared with the healthy subjects, gut microbial diversity was significantly decreased in CKD patients, and the microbial community was remarkably distinguished from the healthy controls. Phyla Bacteroidetes, Proteobacteria, and Actinobacteria were significantly enriched, while Firmicutes and Verrucomicrobia were reduced in the CKD patients versus the healthy controls. Genera *Klebsiella* and *Enterobacteriaceae* were enriched, while *Lactobacillu* and *Lachnospira* were reduced in CKD [24]. Considering disruption in the gut microbiome has been implicated in the pathophysiology of DKD [16], the next step of our study investigated the effects of CK supplementation on compositions of gut microbiota. The analysis of microbial diversity confirmed different composition of microbiota which was clearly separated among the experimental groups in PCoA plot (Figure 2A) and NMDS plot (Figure 2B). In addition, the major composition of microbiota was shown among all groups at phylum and genus levels (Figure 2C). Obviously, Firmicutes, Bacteroidetes and Proteobacteria were the predominant phylum, and *Lactobacillus*, *Bacteroides*, and *Enterococcus* were dominant at the genus level. Two phyla including Bacteroidetes and Proteobacteria were enriched, while phylum Firmicutes was reduced in db/db mice versus db/m mice. Genera *Bacteroides*, *Klebsiella* and *Enterobacteriaceae* were enriched, while *Lactobacillu* and *Akkermansia* were reduced in db/db mice. In particular, the proportion of Firmicutes was 52.8% and Bacteroidetes 42.8% in db/db mice, whereas CK supplementation significantly enhanced the proportion of Firmicutes (72.5%) and reduced the proportion of Bacteroidetes (26.4%) (Figure 2D). Further differences in microbiota were then analyzed by LefSe analysis (Figure 2E) and heatmap analysis (Figure 2F). Combined with analysis of OUT values, we found that CK supplementation was effective not only in upregulating the genera proportion of *Lactobacillu* and *Akkermansia*, but also in downregulating the genera proportion of *Bacteroides*, *Paraprevotella*, *Unidentified Ruminococcaceae*, *Oscillibacter*, *Unidentified Enterobacteriaceae*, *Candidatus Saccharimonas*, *Ruminiclostridium*, *Rikenella*, *Romboutsia*, *Lachnoclostridium*, and *Parasutterella* in comparison with the model group (Figure 2F,G).

### 2.3. CK Supplementation Reduced Microbial Metabolite IMP as a TLR4 Agonist Related to Proteinuria in DKD

**Figure 3:**
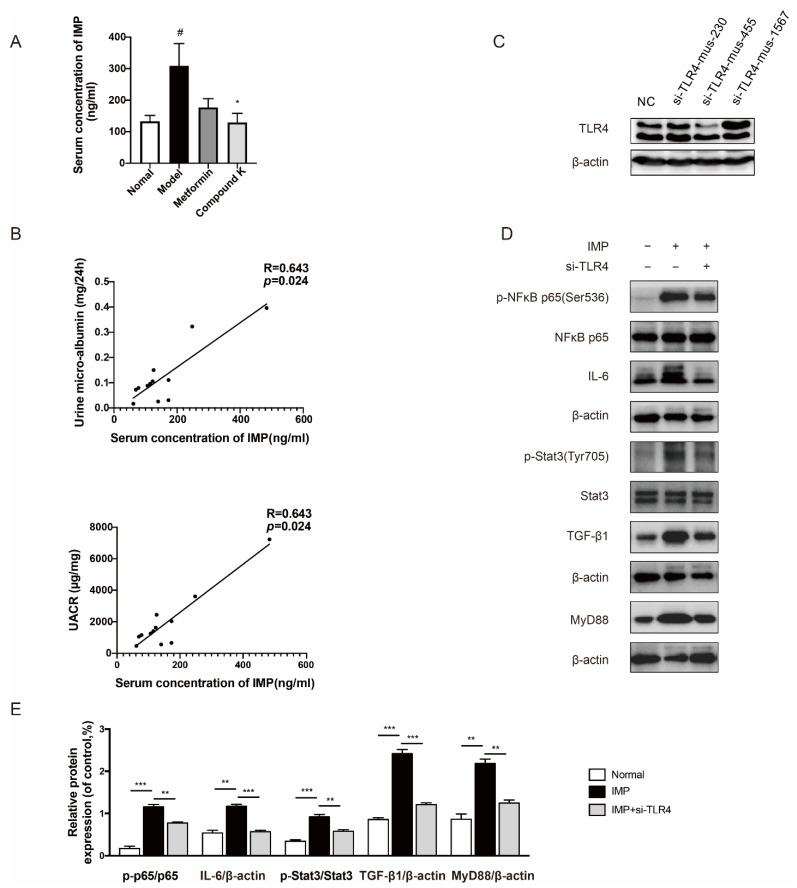
CK supplementation reduced microbial metabolite IMP as a TLR4 agonist related to proteinuria in DKD. (**A**) Serum levels of IMP in mice. Data were expressed as means ± SEM (*n* = 7). ^#^ *p* < 0.05 compared to normal. * *p* < 0.05 compared to model. (**B**) Positive correlation between serum levels of IMP and urine levels of microalbumin and UACR. (**C**) The protein expression of TLR4 was detected with or without siRNA for TLR4. (**D**) The levels of phospho-NF-κB, -NF-κB, IL-6, phospho-Stat3, Stat3, TGF-β1, and MyD88 proteins were detected in mesangial cells by IMP pretreatment with or without siRNA for TLR4 (mus-455). (**E**) Data in (**D**) were expressed as means ± SEM (*n* = 3). NC, negative control. *** *p* < 0.001, ** *p* < 0.01.

Furthermore, dietary and supplementary components not only affect microbial composition but also serve as substrates for many microbial enzymes to change its metabolism [13]. Advances in previous metabolomics have discovered thousands of microbe-derived metabolites, some of which have been detected within host tissues, acting as communicator between host and microbiota. It has been reported that trimethylamine N-oxide (TMAO), polyphenols, protein-bound uremic toxins, and other metabolites had aggravated the progression of DKD [25]. However, there is no evidence to exclude the effect of IMP on DKD. Thus, targeted measurement of IMP in serum of mice was carried out to validate our hypothesis whether IMP increased in DKD subjects. The data showed that IMP was present at higher levels in db/db mice compared to db/m mice, whereas CK supplementation significantly decreased the high level of serum IMP in db/db mice (Figure 3A). We further evaluated a significant positive liner correlation between levels of IMP and renal functional parameters such as urine microalbumin (R = 0.643, *p* < 0.05), and UACR (R = 0.643, *p* < 0.05) in db/db mice (Figure 3B).

Imbalance of gut microbiota also destroys the intestinal barrier to cause abnormal accumulation of uremic toxins in kidneys, and we just identified that IMP played a negative role in the homeostasis of the intestinal barrier [26]. In particular, TLR4 expressed by cells is commonly stimulated by structural motifs characteristically expressed by bacteria, viruses, and fungi known as pathogen-associated molecular patterns (PAMPs) [27,28]. In addition, endogenous molecules can also interact directly or indirectly with TLR4, such as heat-shock proteins, hyaluronic acid, and LPS [9]. We explored whether the effect of IMP on renal injury was relative to TLR4. When silencing TLR4 by specific siRNA (Figure 3C), the simulating effects of IMP on phosphorylation of nuclear factor kappa B (NF-κB) and signal transducer and activator of transcription-3 (Stat3), as well as protein expression of interleukin-6 (IL-6), transforming growth factor β1 (TGF-β1), and myeloid differentiation factor 88 (MyD88) were obviously abolished (Figure 3D,E).

### 2.4. CK Supplementation Presents Inhibiting Effects on TLR4 Signaling Pathway in db/db Mice

**Figure 4:**
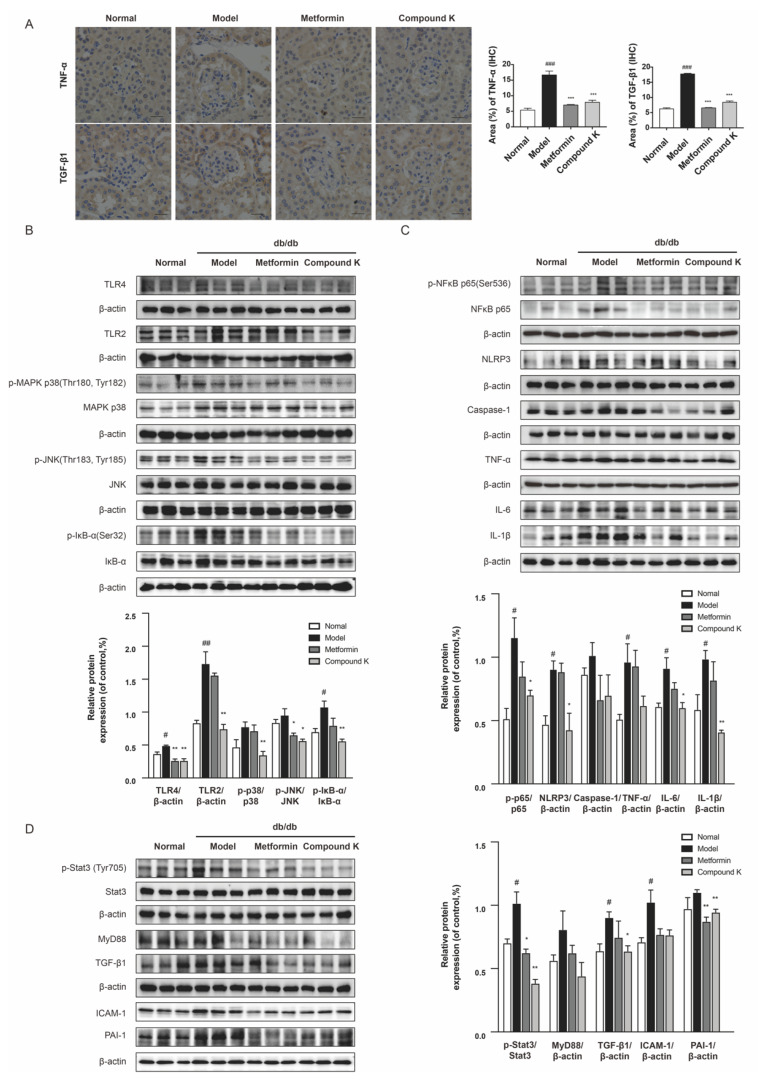
CK supplementation presents inhibiting effects on TLR4 signaling pathway in db/db mice. (**A**) Expression level of TNF-α and TGF-β1 in renal tissue was detected by immunohistochemistry. Scale bar, 20 μm. Relative expression examined by immunohistochemistry was analyzed and shown in histogram. (**B**) The levels of TLR4, TLR2 and phosphorylated and total MAPK p38, JNK and IκB-α proteins. (**C**) The levels of phosphorylated and total NF-κB p65 and levels of NLRP3, caspase 1, TNF-α, IL-6, and IL-1β proteins. (**D**) The levels of phosphorylated and total Stat3 and levels of MyD88, TGF-β1, ICAM-1, and PAI-1 proteins. Data were expressed as means ± SEM (*n* = 7). ^###^ *p* < 0.001, ^##^ *p* < 0.01, ^#^ *p* < 0.05 compared to normal. *** *p* < 0.001, ** *p* < 0.01, * *p* < 0.05 compared to model.

To confirm the renoprotective potency of CK, we next analyzed the changes of key protein expressions in the TLR4 signaling pathway. Subsequently, increases in the expression levels of TNF-α and TGF-β1 were significantly observed by immunohistochemistry staining in the glomeruli of db/db mice, whereas CK supplementation can recover upregulation of such pro-inflammatory and pro-fibrotic proteins (Figure 4A). Our findings also showed that the renal expression level of TLR4 and TLR2, as well as phosphorylation of IκB-α was largely enhanced in db/db mice compared with non-diabetic littermates. A diet supplemented with CK can significantly decrease overexpression of TLR4 and TLR2, and abnormal increases in phosphorylation of mitogen-activated protein kinase (MAPK) p38, c-jun N-terminal kinases (JNK), and inhibitor α of NF-κB (IκBα) in db/db mice (Figure 4B).

Activation of TLR4 signaling contributes to increased production of proinflammatory mediators, and the sustained chronic inflammatory state associated with diabetes [29]. We further examine the role of CK supplementary in NF-κB activated inflammation. As shown in Figure 4C, the phosphorylation of NF-κB was strongly increased in db/db mice, which was markedly relieved by CK. Consistent with NF-κB, the level of inflammasome component nucleotide-oligomerization domain-like receptor 3 (NLRP3) was also suppressed by CK supplementation in db/db mice and its downstream caspase-1 of CK group showed a decreasing tendency. Subsequently, the inhibitory effects of CK on NF-κB effectively suppressed the expression of renal inflammation cytokines, IL-6 and IL-1β.

In addition, there is strong evidence that TLR4 play distinct roles in the pathogenesis of renal fibrosis [27,30]. Hence, we further investigated the functional effects of CK on renal fibrosis in vivo. Our results showed that Stat3 phosphorylation was markedly increased in the kidneys of db/db mice. As expected, CK treatment decreased the increasing phosphorylation of Stat3. We further examined the protein expression for TGF-β1 in experimental groups of renal tissues, showing TGF-β1 expression was upregulated in the kidneys of db/db mice, while CK treatment reduced this upregulation. The significant downregulation of pro-fibrotic markers PAI-1 in db/db mice treated with CK confirmed its anti-fibrotic effect (Figure 4D), which may be relative to the inhibition of TLR4.

### 2.5. CK Suppresses TLR4 Signaling Pathway in the Presence of High Glucose Concentration

**Figure 5:**
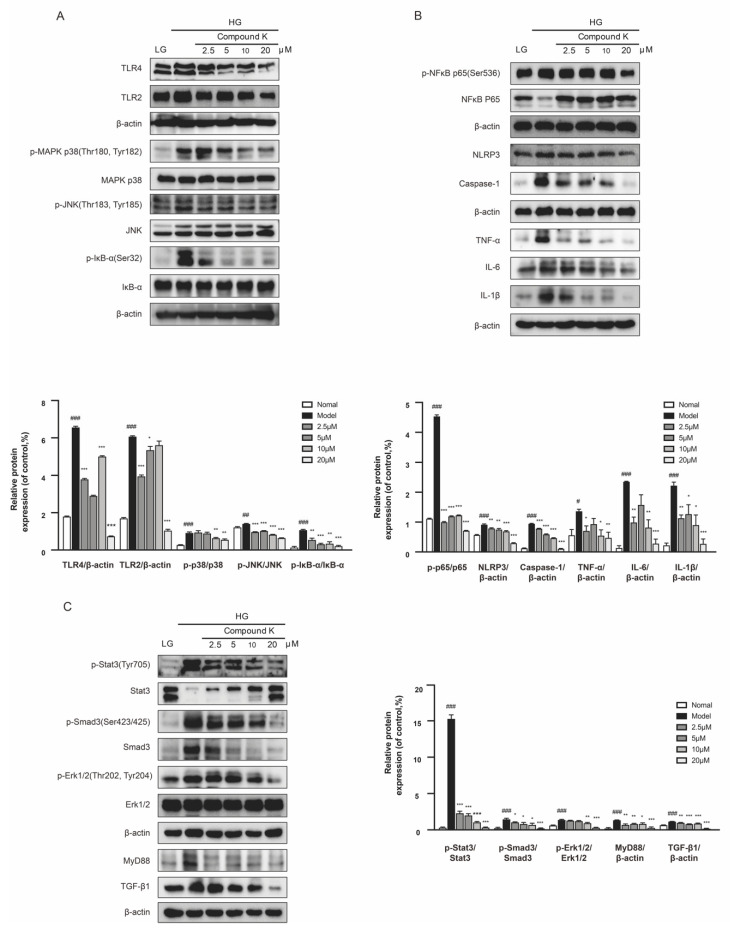
CK suppresses TLR4 signaling pathway in the presence of high glucose concentration. (**A**) The protein expression of TLR4, TLR2 and phosphorylated and total MAPK p38, JNK, and IκB-α were detected in mesangial cells. (**B**) The protein expression of phosphorylated and total NF-κB, and NLRP3, caspase 1, TNF-α, IL-6, and IL-1β were detected in mesangial cells. (**C**) The protein expression of phosphorylated and total Stat3, Smad3, and Erk1/2; the protein expression of MyD88 and TGF-β1 were detected. Data were expressed as means ± SEM (*n* = 3). LG, low glucose medium; HG, high glucose medium. ^###^ *p* < 0.001, ^##^ *p* < 0.01, ^#^ *p* < 0.05 compared to normal. *** *p* < 0.001, ** *p* < 0.01, * *p* < 0.05 compared to model.

As revealed by the in vivo experiment, CK could mitigate not only functional but also pathological damages associated with DKD. Therefore SV40 MES 13 cells were used to assess the mechanisms. The results showed CK treatment suppressed the HG-induced increase in the expression levels of TLR4 and TLR2 proteins, followed by the decreased phosphorylation of MAPK p38, JNK, and IκΒα in the presence of high glucose (HG) concentration (Figure 5A). Because TLR4 exerts its biological activities via both NF-κB-dependent inflammatory response and TGF-β1-dependent fibrotic response, we examined the NF-κB-dependent pathways including NF-κB, NLRP3, caspase 1, TNF-α, IL-6, and IL-1β in SV40 MES 13 cells. CK treatment inhibited NF-κB-mediated inflammation responses (Figure 5B). Then we explored the TGF-β1-dependent fibrotic response. CK significantly attenuated HG-induced increased expression of TGF-β1 and phospho-Smad-3 proteins, along with phospho-Stat3, MyD88, and phospho-extracellular signal-regulated kinase 1/2 (Erk1/2) (Figure 5C).

### 2.6. CK Attenuates DKD Renal Injury through Inhibiting IMP-Induced Inflammation and Fibrosis

**Figure 6:**
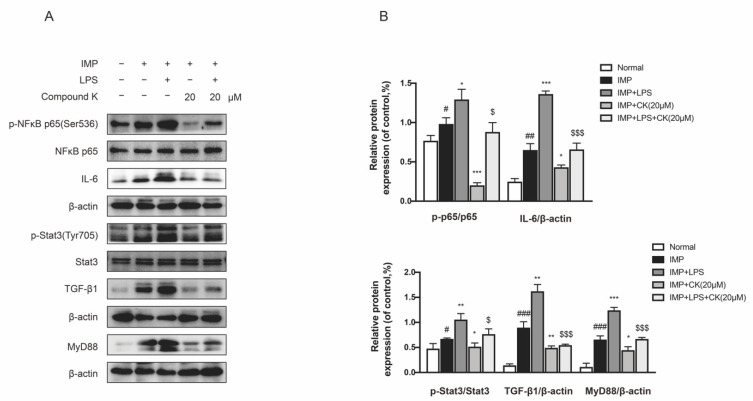
CK attenuates DKD renal injury through inhibiting IMP-induced inflammation and fibrosis. (**A**) The levels of TLR4 pathway related proteins were detected by CK treatment under IMP pretreatment with or without LPS. (**B**) Data in (**A**) were expressed as means ± SEM (*n* = 3). ^###^ *p* < 0.001, ^##^ *p* < 0.01, ^#^ *p* < 0.05 compared to normal group. *** *p* < 0.001, ** *p* < 0.01, * *p* < 0.05 compared to IMP group. ^$$$^ *p* < 0.001, ^$^ *p* < 0.05 compared to IMP + LPS group.

SV40 MES 13 cells were treated with IMP or TLR4 specific agonist LPS to investigate the protective effect of CK related to inhibiting IMP induced injury. We found IMP actually stimulated serious inflammatory and fibrotic responses, showing a significant increased expression of phospho-NF-κB, IL-6, phospho-Stat3, TGF-β1, and MyD88. We also discovered a reduction in CK treatment to the stimulation by IMP. We confirmed IMP accelerated renal dysfunction was dependent on TLR4. We then examined the effects of TLR4 specific agonist LPS on the inhibiting activity of CK with IMP intervention. As a result, CK treatment can reduce the enhancement by LPS and IMP (Figure 6A,B). Collectively, our findings confirmed that CK protected DKD through regulating intestinal microbiota by inhibition of IMP-mediated TLR4 pathways.

## 3. Discussion

As gut–kidney axis theory proposes, recent studies consistently suggest a close relationship between the gut tract and kidneys and material metabolism, immune and inflammation response, intestinal barrier, composition and function of gut microbiota [18,31]. The gut microbiota makes up the largest microecosystem in the human body. When its composition changes, the abnormal accumulation of gut microbial metabolites and endogenous toxins is associated with increasing mortality and morbidity due to loss of renal function. For instance, as it comes to DKD, it commonly appears that *Lactobacillus* and *Akkermansia* reduce, leading to the decrease in short-chain fatty acids (SCFAs) [32]; conversely, *Bacteroides*, *Paraprevotella*, *Oscillibacter*, and *Lachnoclostridium* rise, leading to the increase in TMAO, LPS, phenyl sulfate (PS), and indoxyl sulfate (IS). SCFAs have been reported to have multiple beneficial regulatory roles in DKD through inhibiting oxidative stress and inflammation to recover renal function [25,33]. On the contrary, TMAO, LPS, PS, and IS have been suggested to contribute to renal dysfunction by activating the renin–angiotensin–aldosterone system (RAAS) and the endothelin system, and then inducing insulin resistance, inflammation, oxidative stress, and fibrosis [34,35]. Once the DKD disease process has started, intensive glucose and blood pressure control shows limited benefits on metabolic, inflammatory, and hemodynamic progression, sharing a final common manifestation of excess accumulated connective tissues and renal fibrosis. Given the dual problems of a significant risk of progression from DKD to ESRD and increased cardiovascular morbidity and mortality, it is essential to identify patients at risk of DKD and initiate protective renal therapies in advance.

Ginseng (*Panax Ginseng*) and American ginseng (*Panax Quinquefolius*) have been universally utilized as dietary supplements for centuries [36,37]. Ginseng’s long history in Asia is a testament to its utility for reducing inflammation, benefiting brain function, and boosting the immune system, known as part of traditional Chinese medicine. Traditionally, ginseng was applied for DKD therapy to increase the body’s ability to respond to deficiency of vital energy. Most of the biological activities of ginseng are derived from the predominate constituents, ginsenosides [38,39,40]. Neither ginsenosides nor their metabolites/derivatives have been demonstrated to be exerting activities in insulin resistance, inflammatory responses, oxidative stress, and fibrosis. Of note, CK is a microbially derived metabolite from the protopanaxadiol type ginsenoside [41]. This study used CK supplementation to evaluate changes of DKD related indexes in db/db mice, revealing that early intervention by CK supplementation indeed attenuated microalbuminuria significantly. Pathological manifestations through HE, PAS and Masson staining, indicated that CK supplementation can alleviate the glomerular and tubular basement membrane thickening, mesangial matrix expansion, inflammatory infiltration, and fibrous deposition. The results indicated protecting effects of CK supplementation on renal function.

Furthermore, we assessed the effects of CK on the regulation of gut microbiota in db/db mice. CK supplementation improved the composition from the series analysis at the level of phylum, class, order, family, and genus. In detail, CK supplementation increased properties of *Akkermansia* and *Lactobacillus*, which have demonstrated beneficial effects in anti-inflammation and protecting the gut barrier [32]. By comparison with the current studies, the *Paraprevotella*, *Rikenella*, and *Parasutterella* increases in the CKD cohort can be applied for the distinguishment between individuals with CKD and healthy subjects [24]. *Oscillibacter* was reported to be associated with the production of TMAO, *Bacteroides* was thought to be linked with the production of IMP [42], and *Candidatus Saccharimonas*, *Ruminiclostridium*, *Romboutsia* and *Lachnoclostridium* were confirmed to be connected with the injury of gut barrier [43]. We can speculate that abnormal increases in those microbial genus represent possible renal injury. In concert with these previous studies, our study reported the effects of CK supplementation on decreasing properties of *Bacteroides*, *Paraprevotella*, *Unidentified Ruminococcaceae*, *Oscillibacter*, *Unidentified Enterobacteriaceae*, *Candidatus Saccharimonas*, *Ruminiclostridium*, *Rikenella*, *Romboutsia*, *Lachnoclostridium*, and *Parasutterella* in db/db mice, meaning improving microbial disruption to abolish renal damages.

Interestingly, we previously identified that histidine-derived metabolite IMP can aggravate colitis through impairing the intestinal barrier [26]. Meanwhile, it has been elucidated that a higher plasma concentration of IMP in type 2 diabetic patients, not only was negatively associated with microbial diversity, but also caused glucose intolerance by destroying insulin signaling [42,44]. Presently, there are few reports that study the relationship between IMP and DKD. So, we explored the effects of CK supplementation on renal function around microbial IMP. First, CK supplementation reduced serum concentration of IMP in db/db mice. Second, IMP was positively associated with levels of microalbumin and UACR, similar to PS and IS. Third, IMP can induce renal inflammation in mesangial cells and activate TLR4-mediated signaling via the alternative NF-κB/TGF-β-dependent pathway. Importantly, the renal protein overexpression in TLR4 signaling may be resulted from microbial IMP. Consequently, we also confirmed CK can attenuate IMP-induced abnormal protein expression in vivo and vitro.

## 4. Materials and Methods

### 4.1. Materials

The following items were purchased from the cited commercial sources: CK (molecular weight, 622.87; purity, 98%) from Yuanye Bio-Technology Co., Ltd. (Shanghai, China), metformin (98% purity) from Tixi Aihua Industrial Development Co., Ltd. (Shanghai, China) and IMP from Bachem AG (Bubendorf, Switzerland). The murine SV40 transfected mesangial cell line SV40 MES 13 was acquired from ATCC (Manassas, VA, USA). Small interfering RNA (siRNA) for TLR4 and control siRNA were synthesized by GenePharma Co., Ltd. (Shanghai, China). The siRNA sequence for targeting TLR4 (mus-455) was 5′-GGACAGCUUAUAACCUUAATT-3′. As negative control, a siRNA sequence targeting luciferase was used: 5′-UUCUCCGAACGUGUCACGUTT-3′.

### 4.2. Animals and Experimental Design

Type 2 diabetes male BKS-*Lepr^em2Cd479^*/Gpt mice (db/db mice, 36 ± 2 g, Specific Pathogen Free) and lean wild type littermates (db/m mice, 21 ± 2 g, SPF) aged 7 weeks were obtained from Model Animal Research Center of Nanjing University (License ID: SCXK2019-0006, Nanjing, China). The mice were maintained in controlled laboratory conditions (room temperature at 22 °C, humidity of 60% with a 12 h light/dark cycle) with a standard chow diet and water ad libitum.

After one-week adaption, according to serum glucose level, experimental mice were allocated to the following groups (*n* = 7): wild type mice fed regular chow (normal group), db/db mice fed regular chow (model group), db/db mice fed a diet supplemented with 0.2% metformin (Metformin group), and db/db mice fed a diet supplemented with 0.03% CK (compound K group). The treatment period lasted 16 weeks.

### 4.3. Analysis of Biochemical Parameters

After 16 weeks treatment, the mice were sacrificed by cervical dislocation and blood samples and kidney tissues were collected. Urine biochemical parameters were measured using biochemical kits. UACR was calculated using urinary albumin/creatinine and was expressed as μg/mg.

### 4.4. Morphological Changes

The 10% formalin-fixed kidney was used for histopathological examination including hematoxylin–eosin (HE), periodic acid-Schiff (PAS), and Masson’s trichrome staining. The morphological changes were viewed under the light microscope (400× amplification; BX43, Olympus, Japan). The mesangial matrix index was defined as ratio of mesangial matrix area to glomerular area. Twenty randomly glomeruli in each kidney were selected to evaluate the mesangial matrix index in terms of the severity of glomerular damage (HE). Quantitative analysis of PAS staining was used for the semiquantitative analysis of mesangial expansion. Quantitative analysis of Masson staining was used for the semiquantitative analysis of collagen volume fraction. Histological analysis was performed in a blinded manner utilizing Image J software (NIH, Bethesda, MD, USA).

### 4.5. Microbial Diversity Analysis

Fresh fecal pellets were collected 2 days before the mice were sacrificed. The relative amounts of total bacteria were measured by quantitative real-time PCR based on 16S rRNA gene. The 16S rRNA gene was amplified using the primers 515F (5′-GTGYCAGCMGCCGCGGTAA-3′) and 806R (5′-GGACTACNVGGGTWTCTAA-3′) for further quantification and qualification of PCR products. The differences between samples were analyzed using the Student’s *t*-test in the R software (ver. 3.2.0). Results with *p* value < 0.05 were considered statistically significant.

### 4.6. Metabolite Analysis

According to a previous report [44], for the targeted measurement of imidazole propionate (IMP), mice serum samples were extracted with ice-cold acetonitrile. After a series of derivatization, the samples were evaporated and reconstituted in a mixture of methanol/water (*v*/*v* = 1:1). Samples (5 μL) were injected onto a Kinetex Bi-phenyl 100A column (3 × 50 mm with 2.6 μm particles, Phenomenex) and separated using a gradient consisting of water with 0.1% formic acid (A-phase) and acetonitrile with 0.1% formic acid (B-phase). Mass spectrometric analysis was performed using an SHIMAZDU 30AD LC system (Shimazdu, Kyoto, Japan) coupled to a QTRAP 4500 (AB Sciex, Concord, ON, Canada). The IMP was detected by multiple reaction monitoring using the transition 197/81. Calibration curves of IMP were made in water and treated the same way as the samples.

### 4.7. Immunohistochemistry (IHC) Staining Analysis

Kidney tissues were fixed in 10% formalin, and embedded in paraffin. Immunostaining was conducted in 4-μm paraffin sections with antigen retrieval and protein blocking. The antibodies utilized in the study included TNF-α and TGF-β1. After immunostaining, sections were counterstained with hematoxylin and viewed under the light microscope (400× amplification; BX43, Olympus, Japan). The quantitation of positive staining signals was measured and presented as percentage of the area through Image J software (NIH, Bethesda, MD, USA).

### 4.8. Cell Culture and Treatment

The mesangial cells (passage number between 4 and 10) were cultured in a 3:1 mixture of DMEM and Ham’s F-12 medium containing 5% FBS, and 1% antibiotic/antimycotic solution at 37 °C in a humidified 5% CO_2_ incubator. For the experimental setup, cells were grown to subconfluence and subsequently made quiescent in serum-free low glucose (5.5 mM glucose, LG) DMEM for 24 h before changing to the experimental medium (serum free). Cells were then cultured with high glucose (25 mM glucose, HG) with or without pretreatment with IMP (100 μM) for an additional 24 h. The cells were then transfected with siRNA for TLR4 (100 nM) to explore the potential mechanism of IMP. In addition, different concentrations of compound K (2.5, 5, 10, 20 μM) and lipopolysaccharide (0.5 μg/mL, LPS) were used to examine the effects of CK on HG and IMP-induced changes in mesangial cells.

### 4.9. Western Blot Analysis

The protein analysis was performed by Western blot. Briefly, the protein loading samples (amounts of 40–60 μg) were separated by SDS–PAGE and then transferred and immobilized to a polyvinylidene fluoride membrane. The membranes were washed, blocked, and incubated with analyte-specific primary antibody. Secondary antibodies conjugated with 1:10,000 dilution of the horseradish peroxidase were applied for 1 h. Protein bands were visualized by enhanced chemiluminescence kits and detected and analyzed using a ChemiDoc™ MP Imaging System (Bio-Rad Laboratories, Hercules, California, USA). β-actin was used as a loading control. Results were expressed as the integrated optical density relative to β-actin. Full scans of Western blot assays are shown in Figures S1–S4.

### 4.10. Statistical Analysis

The complete data sets are demonstrated as mean ± SEM. Normality of data were tested by the Shapiro–Wilk test. When *p* > 0.05, null hypothesis accepted and data were normally distributed. Statistical differences between two groups were analyzed using the unpaired Student’s *t*-test. Statistical differences between multiple groups were analyzed using the one-way analysis of variance (ANOVA). Correlation analysis was tested by Spearman correlation analysis (SPSS statistical software, version 26.0). The value *p* < 0.05 was considered statistically significant.

## 5. Conclusions

Our result has successfully highlighted the improving effects of CK supplementation on gut microbiota and serum metabolites in DKD mammal models. As dietary-mediated therapies are gradually becoming more acceptable to patients with DKD, we recommend increasing the intake of CK or a diet containing CK, which might be helpful for early intervention of DKD.

## 6. Patents

A patent “Application of ginsenoside compound K in the preparation of drugs for the treatment of diabetic kidney disease” (Application No. 202110986566.6) is being applied from the work reported in this manuscript.

## Data Availability

All data generated or analyzed during this study are available from the corresponding authors on reasonable request.

## Supplementary Materials

The following supporting information can be downloaded at: https://www.mdpi.com/article/10.3390/ijms232112863/s1.
