# Supplementary material for: Ginsenoside Compound K Ameliorates Development of Diabetic Kidney Disease through Inhibiting TLR4 Activation Induced by Microbially Produced Imidazole Propionate
Source: Int J Mol Sci. 2022 Oct 25;23(21):12863. doi: 10.3390/ijms232112863 (PMC9656537; doi:10.3390/ijms232112863)

Supplementary material for

Ginsenoside Compound K Ameliorates Development of Diabetic Kidney Disease through Inhibiting TLR4 Activation Induced by Microbially Produced Imidazole Propionate

Qian Chen [1,2,†], Dongwen Ren [1,†], Luokun Liu [2], Jingge Xu [2], YuZheng Wu [1], Haiyang Yu [2], Mengyang Liu [2], Yi Zhang [1,*], and Tao Wang [1,2,*]

[1] State Key Laboratory of Component Based Chinese Medicine, Tianjin University of Traditional Chinese Medi-cine, 10 Poyanghu Road, Jinghai District, Tianjin 301617, China

[2] Haihe Laboratory of Modern Traditional Chinese Medicine, Tianjin University of Traditional Chinese Medicine, 10 Poyanghu Road, Jinghai District, Tianjin 301617, China

* Correspondence: zhwwxzh@tjutcm.edu.cn (Y.Z.); wangtao@tjutcm.edu.cn (T.W.);

Tel.: +86-22-59596163 (Y.Z.); +86-22-59596572 (T.W.)

† These authors contributed equally to this work.

Figure Legend for Supplementary Material

Supplementary Figure S1. Full scans of western-blot data were shown in Figure 3. Rectangles delimit cropped areas used in the indicated panels in figure 3. β-actin was used as an internal control.

Supplementary Figure S2. Full scans of western-blot data were shown in Figure 4. Rectangles delimit cropped areas used in the indicated panels in figure 4. β-actin was used as an internal control.

Supplementary Figure S3. Full scans of western-blot data were shown in Figure 5. Rectangles delimit cropped areas used in the indicated panels in figure 5. β-actin was used as an internal control.

Supplementary Figure S4. Full scans of western-blot data were shown in Figure 6. Rectangles delimit cropped areas used in the indicated panels in figure 6. β-actin was used as an internal control.

Supplementary Figure S1.

Figure 3

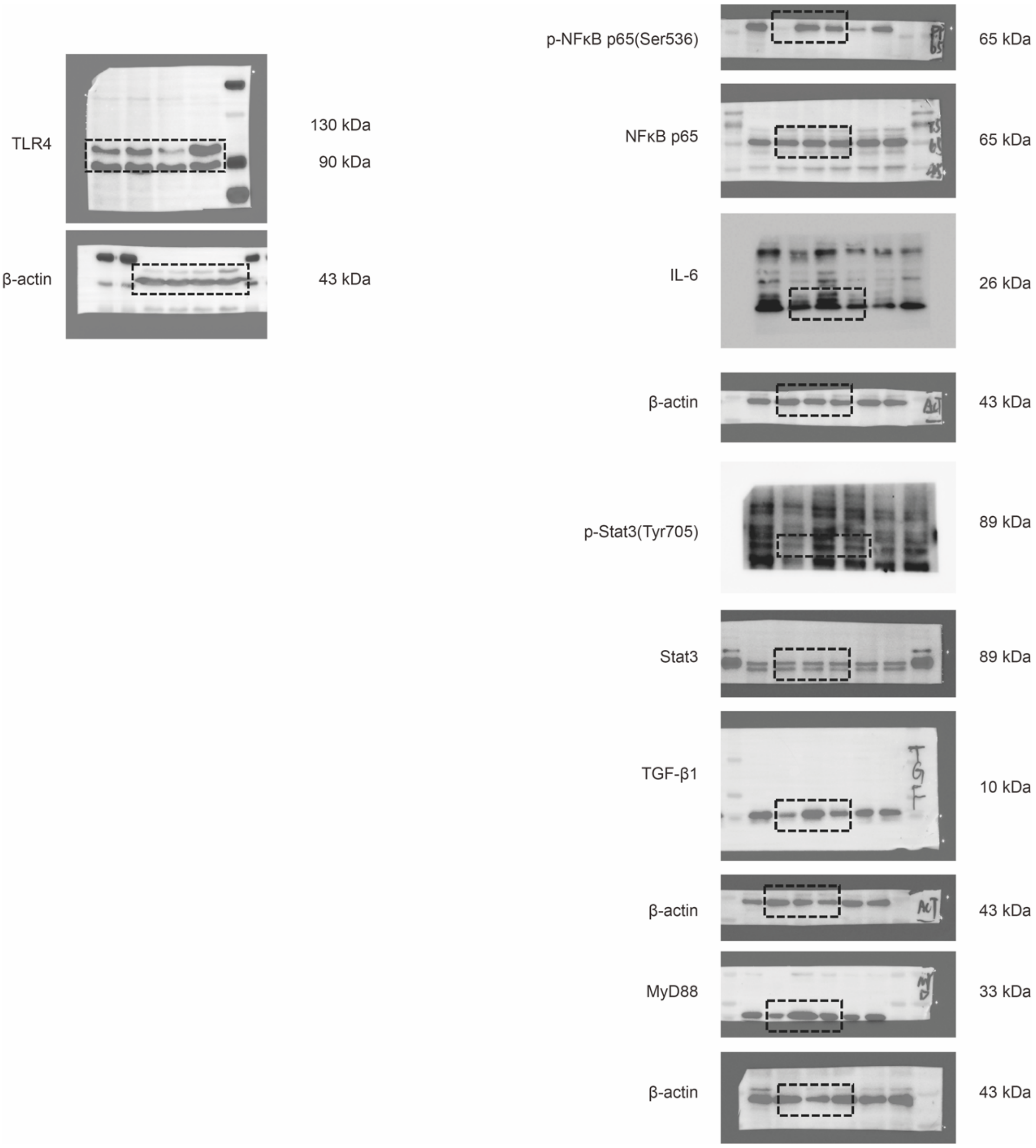

Supplementary Figure S2.

Figure 4

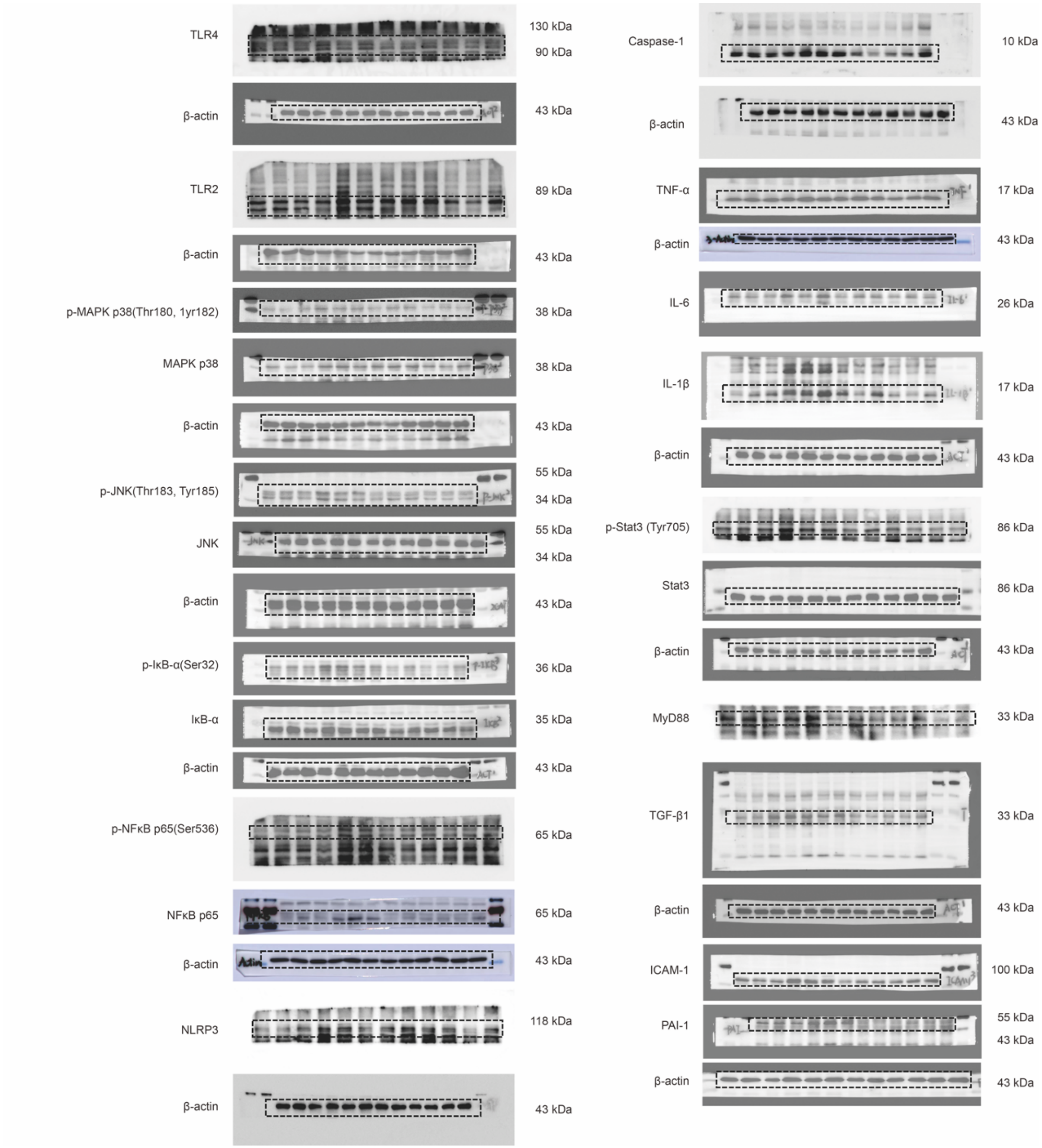

Supplementary Figure S3.

Figure 5

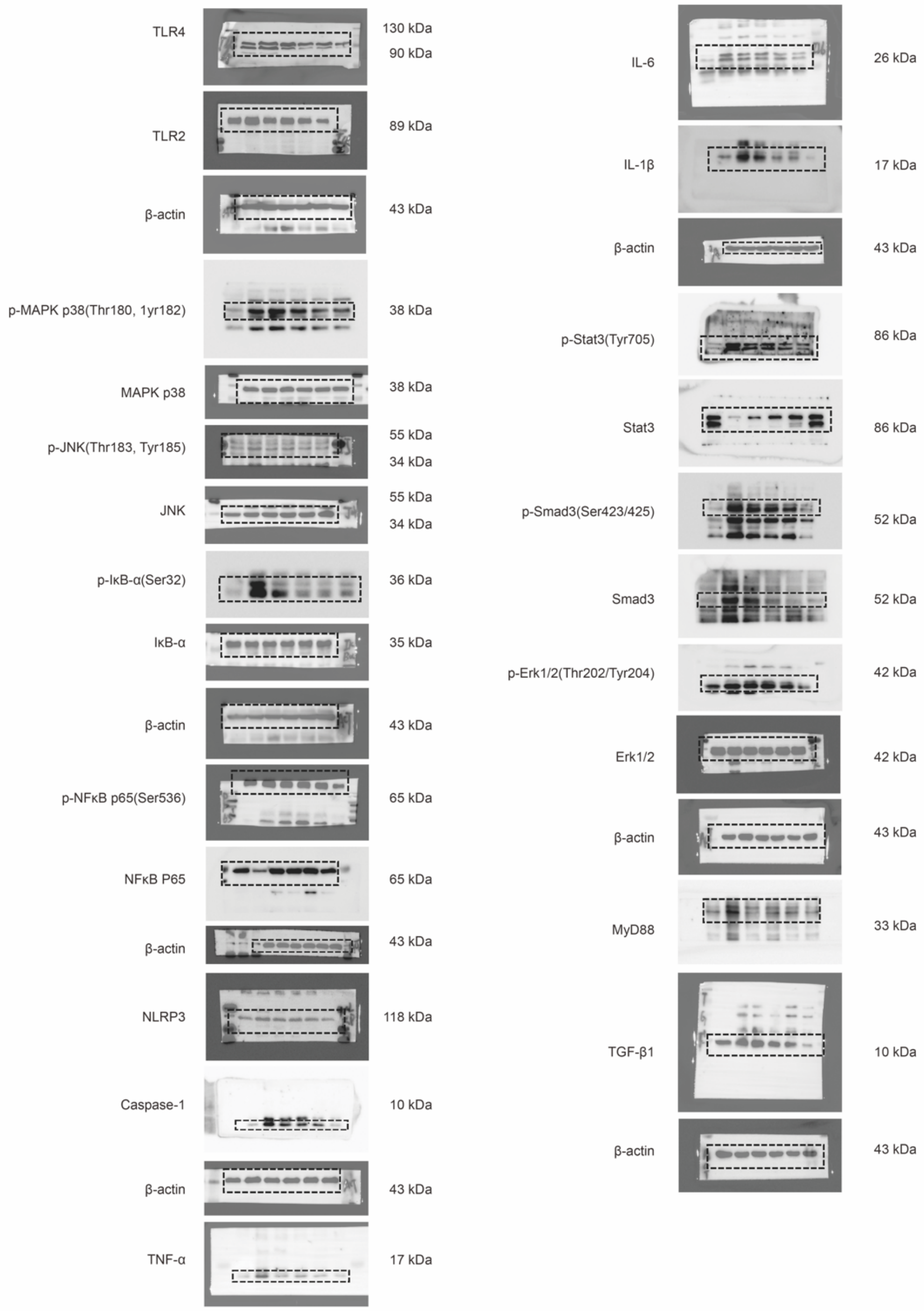

Supplementary Figure S4.

Figure 6

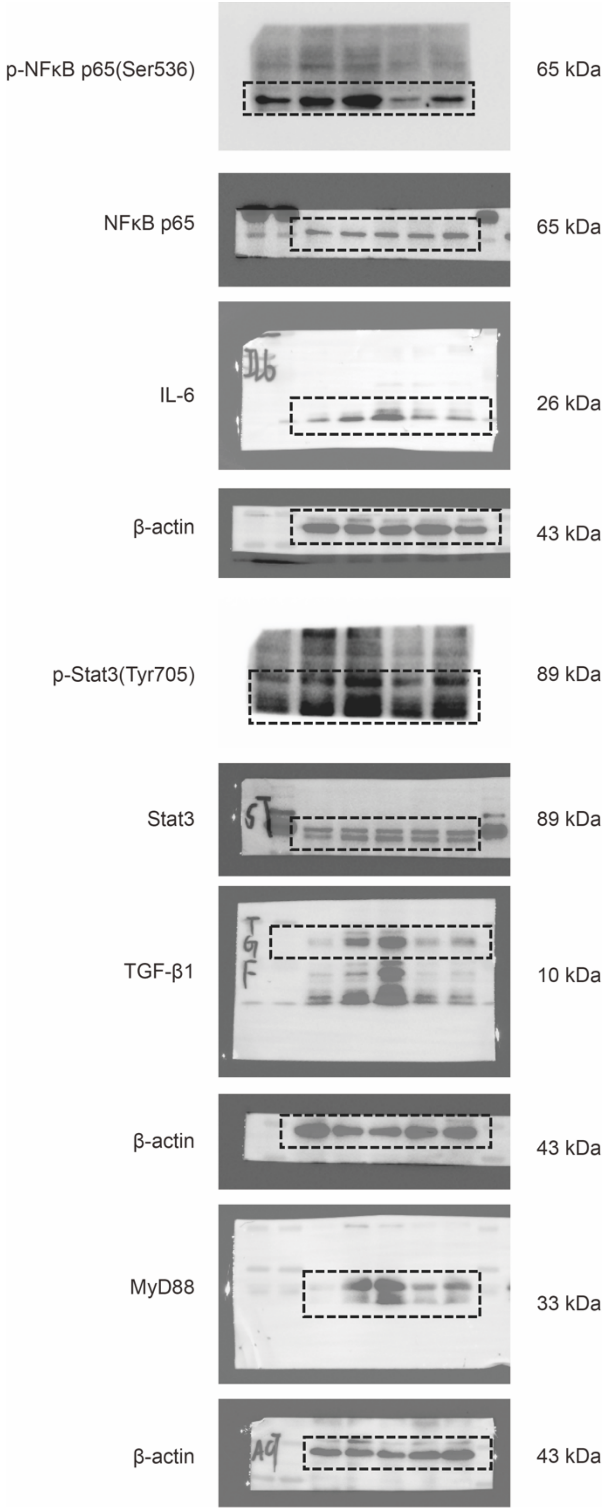

Supplement: Supplementary file 1 [file ijms-23-12863-s001.zip › ijms-1947124-supplementary.pdf]
